# Machine Learning Approach to Inpatient Violence Risk Assessment Using Routinely Collected Clinical Notes in Electronic Health Records

**DOI:** 10.1001/jamanetworkopen.2019.6709

**Published:** 2019-07-03

**Authors:** Vincent Menger, Marco Spruit, Roel van Est, Eline Nap, Floor Scheepers

**Affiliations:** 1 Department of Information and Computing Sciences, Utrecht University, Utrecht, the Netherlands; 2 Department of Psychiatry, University Medical Center Utrecht, Utrecht, the Netherlands; 3 Data Research Office, Antes, Parnassia Group, Rotterdam, the Netherlands

## Abstract

**Importance:**

Inpatient violence remains a significant problem despite existing risk assessment methods. The lack of robustness and the high degree of effort needed to use current methods might be mitigated by using routinely registered clinical notes.

**Objective:**

To develop and validate a multivariable prediction model for assessing inpatient violence risk based on machine learning techniques applied to clinical notes written in patients’ electronic health records.

**Design, Setting, and Participants:**

This prognostic study used retrospective clinical notes registered in electronic health records during admission at 2 independent psychiatric health care institutions in the Netherlands. No exclusion criteria for individual patients were defined. At site 1, all adults admitted between January 2013 and August 2018 were included, and at site 2 all adults admitted to general psychiatric wards between June 2016 and August 2018 were included. Data were analyzed between September 2018 and February 2019.

**Main Outcomes and Measures:**

Predictive validity and generalizability of prognostic models measured using area under the curve (AUC).

**Results:**

Clinical notes recorded during a total of 3189 admissions of 2209 unique individuals at site 1 (mean [SD] age, 34.0 [16.6] years; 1536 [48.2%] male) and 3253 admissions of 1919 unique individuals at site 2 (mean [SD] age, 45.9 [16.6] years; 2097 [64.5%] male) were analyzed. Violent outcome was determined using the Staff Observation Aggression Scale–Revised. Nested cross-validation was used to train and evaluate models that assess violence risk during the first 4 weeks of admission based on clinical notes available after 24 hours. The predictive validity of models was measured at site 1 (AUC = 0.797; 95% CI, 0.771-0.822) and site 2 (AUC = 0.764; 95% CI, 0.732-0.797). The validation of pretrained models in the other site resulted in AUCs of 0.722 (95% CI, 0.690-0.753) at site 1 and 0.643 (95% CI, 0.610-0.675) at site 2; the difference in AUCs between the internally trained model and the model trained on other-site data was significant at site 1 (AUC difference = 0.075; 95% CI, 0.045-0.105; *P* < .001) and site 2 (AUC difference = 0.121; 95% CI, 0.085-0.156; *P* < .001).

**Conclusions and Relevance:**

Internally validated predictions resulted in AUC values with good predictive validity, suggesting that automatic violence risk assessment using routinely registered clinical notes is possible. The validation of trained models using data from other sites corroborates previous findings that violence risk assessment generalizes modestly to different populations.

## Key Points

**Question:**

To what extent can inpatient violence risk assessment be performed by applying machine learning techniques to clinical notes in patients’ electronic health records?

**Findings:**

In this prognostic study, machine learning was used to analyze clinical notes recorded in electronic health records of 2 independent psychiatric health care institutions in the Netherlands to predict inpatient violence. Internal predictive validity was measured using areas under the curve, which were 0.797 for site 1 and 0.764 for site 2; however, applying pretrained models to data from other sites resulted in significantly lower areas under the curve.

**Meaning:**

The findings suggest that inpatient violence risk assessment can be performed automatically using already available clinical notes without sacrificing predictive validity compared with existing violence risk assessment methods.

## Teaser

This prognostic study develops and validates a multivariable prediction model for assessing inpatient violence risk, using machine learning techniques applied to clinical notes written in patients’ electronic health records.

## Introduction

Violence in psychiatric inpatient wards remains a significant problem. A study^1^ combining data from 35 sites worldwide shows 14% to 20% of patients commit at least 1 act of violence during inpatient treatment, and surveys^2^ consistently show most practitioners being affected by violence at some point during their career. Adverse effects on both patients’ and caregivers’ well-being, such as injury, low morale, and high absentee levels, are well known.^3,4^

As an important part of managing inpatient violence, structured violence risk assessment (VRA) instruments have been proposed on the basis of a combination of static and dynamic risk factors. Their predictive validity surpasses that of unstructured clinical judgment, and a reasonable adoption in practice has been achieved, with more than half of all risk assessments performed using an instrument.^5^ However, meta-analyses reveal that only a small subset of risk factors for violent behavior generalize to different populations,^6,7^ and VRA instruments are consequently limited by the robustness of the individual factors that compose them.^8,9^ In addition, the time needed to perform a structured assessment, ranging from minutes to hours, has been identified as an obstacle for daily practice. Although adopting a VRA instrument diminished the number of violent incidents in 1 randomized clinical trial,^10^ other research^11,12^ suggests that its benefits in practice are still moderate because of its limitations.

Developing a prognostic model based on textual data registered in patients’ electronic health records (EHRs) might offer a novel approach to improve VRA. The fact that these data are unstructured and originally designated for treatment presents methodologic challenges but also opportunities in combating selection bias and exploring new associations.^13^ *Machine learning*, a term that refers to a set of statistical techniques that learn from large and potentially noisy data sets, is eminently well suited for this kind of task. Prognostic models obtained using these techniques are automatically tailored to the relevant population and can be fitted in the care process without imposing additional administrative load, circumventing drawbacks of structured VRA instruments. Although many fields of medicine have seen convincing cases of algorithms aiding clinical decision making (eg, cardiology,^14^ dermatology,^15^ and oncology^16^), the field of psychiatry still seems only on the verge of transforming in this direction.^17,18^ In this prognostic study, we tested to what extent textual data from the EHR can be used to automatically assess violence risk by developing and validating multivariable prediction models based on routinely collected clinical notes from 2 independent psychiatric treatment centers in the Netherlands.

## Methods

In this study, we used data extracted from EHRs of 2 independent psychiatric treatment centers in the Netherlands. Data sources were not connected to each other or to sources outside the separate hospitals. We used deidentified data sets by deidentifying clinical notes using the Deindentification Method for Dutch Medical Text (DEDUCE) method.^19^ Demographic variables were limited to sex, year of birth, and *Diagnostic and Statistical Manual of Mental Disorders* (Fourth Edition) diagnosis. The study was reviewed and approved by the University Medical Center Utrecht ethical committee. The committee assessed that obtaining informed consent retroactively was not necessary because of the retrospective nature of the study, the number of participants, the fact that no extra data were obtained, and the use of deidentified data. This report follows the Transparent Reporting of a Multivariable Prediction Model for Individual Prognosis or Diagnosis (TRIPOD) reporting guideline^20^ and Reporting Guidance for Violence Risk Assessment Predictive Validity Studies (RAGEE).^21^

### Cohort Definition

Site 1, used for internal method validation, was the psychiatry department of the academic medical center in Utrecht, the Netherlands. It delivers both secondary and tertiary care in 4 closed short-term treatment wards, including an acute ward and wards that focus on treatment of patients with psychotic disorders, mood disorders, and developmental disorders. A new admission was registered both when a new patient was admitted and when a patient was transferred between psychiatric wards. We allowed an absence of 2 weeks at most during admission, such as for discharge and readmission or temporary admission in a nonpsychiatric department; longer absences were registered as a new admission. Admissions in the developmental disorder ward were excluded according to patient age and the nature of violence. All admissions in other wards that started between January 2013 and August 2018 were included in the data set. We defined no exclusion criteria according to diagnosis, comorbidity, or other psychopathological conditions to maximize the translational value of predictive models. The resulting data set consisted of 3201 admissions of 2211 unique patients.

Site 2, used for external method validation, was a general psychiatric hospital that delivers secondary care, with an additional focus on addiction care. It consists of 47 treatment wards in the area of Rotterdam, the Netherlands. To match the original data set, admissions to 2 forensic psychiatric wards, 25 long-term care wards, and 9 wards that exclusively offer addiction care were not included in the study. All admissions in the 11 retained wards that started between June 2016 and August 2018 were included in the data set. Other conditions were kept equal. The resulting data set consisted of 3277 admissions of 1937 unique patients. Details explaining how data sets from both sites were extracted from EHR systems and how data quality was secured are shown in eAppendix 1 in the Supplement. We did not merge data sets but used the data set from site 1 for developing a machine learning approach, then used the data set from site 2 for externally validating this approach, and finally exchanged trained models between the sites.

### Data Selection

Clinical notes that were written by psychiatrists and nurses were directly extracted from patients’ EHRs. We hypothesized that free text contains information that cannot easily be captured in structured form (eg, behavioral cues or social interactions) yet is relevant for VRA. Notes that were written in the 4 weeks before admission up to the first 24 hours of admission were included in the data sets. Admissions with fewer than 100 words registered after 24 hours (12 admissions in site 1 and 24 admissions in site 2) were excluded from the data set.

### Outcome Variable

Reports of violent incidents were used to determine the outcome for each admission. In both sites mandatory reporting of all violent incidents takes place, including patient-staff and patient-patient violence. On the incident form, staff members who were involved in the incident were required to fill in structured information, a textual description of the incident, and incident severity as measured by the Staff Observation Aggression Scale–Revised.^22^ Our definition of a violent incident included all threatening and violent behavior of a verbal or physical nature directed at another person but excluded self-harm and inappropriate behavior, such as substance use, sexual intimidation, or vandalism. A positive outcome was defined as the presence of at least 1 incident in the first 4 weeks of admission, excluding the first 24 hours. No distinction in incident severity was made.

### Exploratory Analysis

To examine the potential predictive power hidden in clinical notes, we extracted the 1000 most frequent terms in the clinical notes, including bigrams, as binary variables. We then assessed the strength of each term’s association with the outcome using a χ^2^ test and computed Matthews correlation coefficients to obtain the direction of the association. We selected the top 10% of predictors on the basis of their χ^2^ scores in 1000 repeated samples with replacement, computing the fraction of times a term was included among the top predictors as a measure of within–data set generalizability of predictors.

### Machine Learning Models

We used a machine learning approach to perform VRA. Machine learning algorithms are able to detect patterns, if present, in historical data, and a prediction of the future course of treatment can be made on the basis of those patterns. Such an approach applied to textual data must comprise 2 steps: transforming clinical notes into a suitable numerical representation and subsequently feeding these numerical representations into a classification model.

To transform the clinical notes into a numerical form, we used the novel paragraph2vec algorithm,^23^ which learns an accurate numerical representation from a large corpus of text in an unsupervised way (ie, unrelated to outcome). This algorithm, founded in deep learning theory, is capable of using not only verbatim words in a text to determine a representation but also word order and the context of words such as negations. In previous work,^24^ we have shown the added value of this technique over a traditional bag-of-words approach when applied to VRA. The model was trained using a large internal set of clinical notes (ie, not only notes relevant for assessment), with model settings based on available literature without optimization (eAppendix 2 and eTable 1 in the Supplement).

The numerical representations of text were subsequently fed into a support vector machine with a radial kernel,^25^ a model that has previously been shown as appropriate for text classification.^26^ It works by first mapping data points to a higher-dimensional space and then inferring a decision boundary that maintains a maximum margin to these data points. New data points are subsequently classified according to the side of the boundary on which they lie.

### Statistical Analysis

Model training and estimation of model predictive validity were done in a nested cross-validation setup, ensuring that admissions used for learning models were never used to simultaneously determine predictive validity. Different admissions of the same patient were additionally never split over different folds to ensure that predictions were not influenced by information from future admissions of the same patient. The final area under the curve (AUC) was computed by averaging the AUCs of the 5 outer cross-validation folds, while CIs and SEs were established using the method of DeLong et al.^27^ Additionally, performance metrics, such as sensitivity, specificity, and relative risk, were computed by pooling predictions over folds.^28^ The experimental setup is detailed further in eAppendix 3 in the Supplement. After finalizing the results in site 1, an external validation of the machine learning approach was performed in site 2 by training a new model with equal experimental setup. To further elucidate model performance, we investigated predictive validity for early-violence vs late-violence and short-admission vs long-admission subgroups. Finally, trained models were exchanged between sites to test their generalizability.

For the tokens discovered in exploratory analysis, the association with the outcome was determined using a χ^2^ test with a Holm-Bonferroni correction to control the familywise error rate. Differences in AUCs between various internal and external validations were tested for significance using the method of DeLong et al^27^ and Robin et al.^29^ We used a paired test when comparing 2 models on the same data set (ie, when comparing the cross-validated assessment and assessment using a pretrained model) to account for correlation between the 2 AUCs. In all other cases we used an unpaired test. All statistical significances in this study were assessed using 2-sided tests, and *P* < .01 was considered significant. The code for machine learning and statistical analysis was developed in Python software version 3.6 (Python Software Foundation) and is publicly available (eAppendix 4 in the Supplement).

### Qualitative Evaluation

After finalizing the method and results in both sites, a qualitative evaluation was conducted in a focus group with participants, including practitioners, data analysts, and patient representatives from both sites. Participants discussed the method as presented by a researcher (V.M.) and interpreted the results. The participants’ attitude toward the method was positive, and its translation between sites was deemed appropriate. No changes were introduced to the study as a result of the focus group.

## Results

### Data Sets

The final data sets (Table 1) consisted of 3189 admissions from 2209 unique patients in site 1 and 3253 admissions from 1919 unique patients in site 2. Populations differed in age (mean [SD] age, 34.0 [16.6] and 45.9 [16.6] years, respectively), sex (1536 [48.2%] and 2097 [64.5%] men, respectively), and distribution of diagnoses. In both sites, the most commonly occurring diagnosis was schizophrenia or other psychotic disorders, followed by mood disorders and personality disorders in site 1 and substance-related disorders and bipolar disorders in site 2. Similar median (interquartile range [IQR]) lengths of stay (16.0 [6.0-41.0] and 15.0 [5.0-40.5] days), median (IQR) length of clinical notes (2091 [1541-2981] and 1961 [1160-3060] words), and admissions with a violent incidence (290 [9.1%] and 247 [7.7%]) were registered in both sites.

**Table 1.** Descriptive Statistics of the Data Sets Obtained From the 2 Sites

Characteristic	No. (%)	
	Site 1	Site 2
Demographic characteristics		
Age, mean (SD), y	34.0 (16.6)	45.9 (16.6)
Men	1536 (48.2)	2097 (64.5)
Data set		
Admissions, No.	3189	3253
Unique patients, No.	2209	1919
Length of stay, median (IQR), d	16.0 (6.0-41.0)	15.0 (5.0-40.5)
No. of words in notes, median (IQR)	2091 (1541-2981)	1961 (1160-3060)
Admissions with violent incidents	290 (9.1)	247 (7.7)
Incidents		
During admission, No.	962	652
During first 4 wk	658 (68.4)	318 (48.8)
During first 24 h	90 (9.4)	42 (6.4)
Staff Observation Aggression Scale–Revised score, median (IQR) [range]	12.0 (8.0-16.0) [2-21]	11.0 (7.0-14.0) [2-19]
*Diagnostic and Statistical Manual of Mental Disorders* diagnosis^a^		
Anxiety disorder	92 (2.9)	63 (1.9)
Bipolar disorder	65 (2.0)	170 (5.2)
Delirium, dementia, amnesia, and other cognitive disorders	20 (0.6)	109 (3.4)
Depressive disorder	106 (3.3)	150 (4.6)
Developmental disorder	180 (5.6)	29 (0.9)
Eating disorder	57 (1.8)	10 (0.3)
Mood disorder	580 (18.2)	10 (0.3)
Personality disorder	214 (6.7)	116 (3.6)
Substance-related disorder	99 (3.1)	373 (11.5)
Schizophrenia or other psychotic disorder	860 (27.0)	685 (21.1)
None within 12 wk	795 (24.9)	1392 (42.8)
Other	121 (3.8)	146 (4.5)

Abbreviation: IQR, interquartile range.

^a^ Percentage relative to the total number of admissions.

### Machine Learning Models

Several performance metrics of predictive validity, both for in-site validation using nested cross-validation and for other-site validation of pretrained models, were computed (Table 2). Optimal hyperparameters are shown in eTable 2 in the Supplement. An optimal AUC of 0.797 (95% CI, 0.771 to 0.822) was achieved for the internal validation of the method in site 1, while the optimal AUC for the external validation of the method in site 2 was 0.764 (95% CI, 0.732 to 0.797) (Figure). The difference in internal cross-validation AUCs between the 2 sites was not significant (AUC difference = 0.032; 95% CI, −0.009 to 0.074; *P* = .12). Specificity (ie, prediction in the negative class) of models was higher (0.935 to 0.947) than sensitivity (ie, prediction in the positive class; 0.334 to 0.336). The relative risk of violent outcome for patients with predicted high risk vs low risk was 5.121 (95% CI, 4.109-6.330) in site 1 and 6.297 (95% CI, 4.956-7.922) in site 2.

**Table 2.** Predictive Validity of Prognostic Models in Both Sites and Both Internally and Externally Trained

Evaluation	Internal Cross-validation		External Model	
	Site 1	Site 2	Site 1	Site 2
Model evaluated in site	1	2	1	2
Model trained in site	1	2	2	1
AUC (95% CI) [SE]	0.797 (0.771-0.822) [0.013]	0.764 (0.732-0.797) [0.017]	0.722 (0.690-0.753) [0.016]	0.643 (0.610-0.675) [0.017]
Admissions, No.	3189	3253	3189	3253
Negative, No. (%)				
True	2711 (85.0)	2847 (87.5)	2682 (84.1)	2793 (85.9)
False	193 (6.1)	164 (5.0)	218 (6.8)	214 (6.6)
Positive, No. (%)				
True	97 (3.0)	83 (2.6)	72 (2.3)	33 (1.0)
False	188 (5.9)	159 (4.9)	217 (6.8)	213 (6.5)
Specificity (95% CI)	0.935 (0.930-0.940)	0.947 (0.943-0.951)	0.925 (0.921-0.930)	0.929 (0.926-0.933)
Sensitivity (95% CI)	0.334 (0.287-0.383)	0.336 (0.285-0.389)	0.248 (0.205-0.296)	0.134 (0.097-0.179)
Relative risk (95% CI)	5.121 (4.109-6.330)	6.297 (4.956-7.922)	3.314 (2.581-4.214)	1.885 (1.305-2.673)

Abbreviation: AUC, area under the curve.

**Figure.**
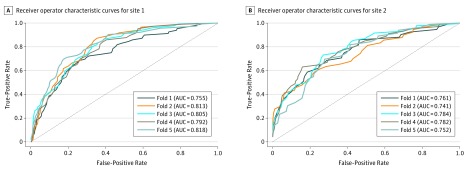
Receiver Operator Characteristic Curves for Internal Cross-validations Receiver operator characteristic curves are shown for each fold, according to internal cross-validation in site 1 (A) and site 2 (B). Dashed diagonal lines denote an area under the curve (AUC) of 0.5, ie, predictive validity equivalent to chance. AUC indicates area under the curve.

The validation of pretrained models in the other site resulted in AUCs of 0.722 (95% CI, 0.690-0.753) in site 1 and 0.643 (95% CI, 0.610-0.675) in site 2. The difference in AUCs between the internally trained model and the model trained on other-site data was significant both in site 1 (AUC difference = 0.075; 95% CI, 0.045-0.105; *P* < .001) and site 2 (AUC difference = 0.121; 95% CI, 0.085-0.156; *P* < .001). Although specificity was still similar, both sensitivity and relative risk were lower compared with in-site validations.

We examined model performance in assessing early vs late violence and violence during short vs long admissions. In both internal and external validations and in both sites, predictive validity was higher for early violence than for late violence as well as higher for short admissions than for long admissions. However, the difference was never significant. For example, for the internal validation in site 1, the difference in AUCs for assessing early violence vs late violence was 0.046 (95% CI, −0.003 to 0.094; *P* = .06), and the difference in AUCs for assessing violence during short admissions vs long admissions was 0.012 (95% CI, −0.041 to 0.066; *P* = .65). Full subgroup analysis is included in eTable 3 and eTable 4 in the Supplement.

### Exploratory Analysis

Of the 1000 most frequent terms from clinical notes, the top 20 terms by generalizability within a data set were selected (Table 3).^30^ Several terms, such as *aggressive*, *angry*, *verbal*, *threatening*, and *irritated*, can directly be associated with violence, whereas other terms, such as *reacts*, *walks*, and *speaks*, describe behavioral cues that may indirectly be associated with violence. The terms *aggressive* and *walked* and their synonyms are seen in both sites. Other terms do not directly co-occur in both sites but have a counterpart with a similar meaning (eg, *colleague* vs *staff* and *door* vs *office)*. All terms generalize well within the data set, being chosen among the top 10% in repeated sampling at least 95% of the time. In site 1, the terms *aggressive*, *reacts*, and *offered* generalize best within the data set, whereas in site 2 the terms *verbal*, *threatening*, and *aggression* compose the top 3. The 47 terms in site 1 and 21 terms in site 2 with highest χ^2^ scores were significantly associated with the outcome after applying a Holm-Bonferroni correction. Matthews correlation coefficients ranged from −0.14 to 0.17, showing weak correlations. Most terms had a positive correlation with violent outcome, except *status voluntary* and *dejected* in site 1, which were negatively correlated with violent outcome (status voluntary: Matthews correlation coefficient, −0.12; 95% CI, −0.14 to −0.09; *P* < .001; dejected: Matthew correlation coefficient, −0.14; 95% CI, −0.17 to −0.11; *P* < .001).

**Table 3.** Results of Exploratory Analysis

Rank^a^	Site 1				Site 2			
	Term (English Translation)^b^	Ratio	MCC (95% CI)^c^	*P* Value^d^	Term (English Translation)^b^	Ratio	MCC (95% CI)^c^	*P* Value^d^
1	Agressief (aggressive)	1.00	0.17 (0.13 to 0.21)	<.001	Verbaal (verbal)	1.00	0.14 (0.10 to 0.18)	<.001
2	Reageert (reacts)	1.00	0.15 (0.11 to 0.19)	<.001	Dreigend (threatening)	1.00	0.13 (0.08 to 0.16)	<.001
3	Aangeboden (offered)	1.00	0.14 (0.11 to 0.18)	<.001	Agressie (aggression)	1.00	0.15 (0.11 to 0.17)	<.001
4	Boos (angry)	1.00	0.16 (0.12 to 0.19)	<.001	Hierop ([up]on this)	1.00	0.13 (0.09 to 0.16)	<.001
5	Deur (door)	1.00	0.14 (0.10 to 0.18)	<.001	Kantoor (office)	1.00	0.12 (0.08 to 0.16)	<.001
6	Loopt (walks)	1.00	0.15 (0.11 to 0.18)	<.001	Personeel (staff)	1.00	0.12 (0.07 to 0.16)	<.001
7	Ibs (arrest)	1.00	0.14 (0.10 to 0.17)	<.001	Aangesproken (spoke to)	1.00	0.11 (0.08 to 0.15)	<.001
8	Aanbieden (offer)	1.00	0.12 (0.08 to 0.15)	<.001	Agressief (aggressive)	0.99	0.11 (0.08 to 0.15)	<.001
9	Noodmedicatie (emergency medication)	0.99	0.14 (0.10 to 0.17)	<.001	Gevaar agressie (danger aggression)	0.99	0.11 (0.07 to 0.15)	<.001
10	Liep (walked)	0.99	0.12 (0.08 to 0.16)	<.001	Agitatie (agitation)	0.99	0.11 (0.07 to 0.14)	<.001
11	Agressie (aggression)	0.99	0.13 (0.09 to 0.18)	<.001	Geirriteerd (irritated)	0.99	0.10 (0.06 to 0.14)	.001
12	Vraagt (asks)	0.99	0.13 (0.10 to 0.17)	<.001	Separeer (seclusion room)	0.99	0.10 (0.06 to 0.15)	<.001
13	Status vrijwillig (status voluntary)	0.99	−0.12 (−0.14 to −0.09)	<.001	Loopt (walks)	0.99	0.11 (0.08 to 0.14)	.02
14	Psychotisch (psychotic)	0.98	0.12 (0.09 to 0.16)	<.001	Grond (ground)	0.98	0.10 (0.06 to 0.14)	<.001
15	Collega (colleague)	0.98	0.11 (0.07 to 0.15)	<.001	Aanvang (commencement)	0.98	0.11 (0.08 to 0.14)	.01
16	Spreekt (speaks)	0.97	0.12 (0.08 to 0.15)	<.001	Mede (also)	0.98	0.10 (0.07 to 0.14)	.001
17	Gehouden (obliged)	0.97	0.11 (0.07 to 0.15)	<.001	Dhr wilde (Mr wanted)	0.98	0.10 (0.06 to 0.14)	.001
18	Beoordelen (judge), verb	0.96	0.11 (0.07 to 0.15)	<.001	Liep (walked)	0.98	0.10 (0.06 to 0.14)	.006
19	Momenten (moments)	0.96	0.12 (0.08 to 0.15)	<.001	Geagiteerd (agitated)	0.96	0.10 (0.06 to 0.14)	.01
20	Somber (dejected)	0.95	−0.14 (−0.17 to −0.11)	<.001	cvd (not available)	0.96	0.10 (0.06 to 0.14)	.004

Abbreviation: MCC, Matthews correlation coefficient.

^a^ The top 20 terms with highest within–data set generalizability (ratio) are included.

^b^ The *Van Dale Dutch–English Dictionary*, 3rd edition,^30^ was used for translations.

^c^ Matthews correlation coefficient is computed to assess the direction of association between the term and outcome.

^d^ *P *values derived from χ^2^ test, and a Holm-Bonferroni correction was applied to obtain corrected *P* values.

## Discussion

To our knowledge, this is the first time that readily available clinical notes from patients’ EHRs were used to assess inpatient violence risk. We applied machine learning techniques to retrospective textual data, to train a model that differentiates patients who show violent behavior during the first 4 weeks of admission from patients who do not. As far as we know, no study has performed VRA using clinical text, and no study has tested automatic VRA in multiple sites. The AUCs of internally cross-validated predictions (0.797 and 0.764) from this study lie in the range that can be seen as acceptable for application in practice. Although in-site validation of models obtained good results, other-site validation of pretrained models resulted in significantly lower predictive validity, corroborating previous findings that VRA generalizes modestly over different populations. This strengthens the case for using locally developed and/or trained models and methods for VRA. Our choice to balance between false-positive and false-negative findings for reporting outcomes resulted in higher predictive validity in the low-risk class (eg, sensitivity) than in the high-risk class (eg, specificity), which is largely in line with existing VRA research. To our knowledge, no assessment method has shown both high sensitivity and high specificity, characterizing the difficulty of performing VRA and the need for further improvements.

Violence risk assessment is a research topic that has been thoroughly described, and the predictive validity of many existing methods, such as VRA checklists and unstructured clinical judgment, has been reported in literature. Although our study, based on other data sets, does not allow making strong claims about whether machine learning improves predictive validity,^31^ we note that our internally validated predictive validities of AUC = 0.797 and AUC = 0.764 lie in the same range of existing methods while overcoming some of their drawbacks. For example, a study by Fazel et al^32^ assessed median (IQR) predictive performance of the 4 most commonly used VRA instruments over 30 different studies (AUC = 0.72 [0.68-0.78]), while another study by Teo et al^33^ assessed the level of accuracy of psychiatric residents (AUC = 0.52) and trained psychiatrists (AUC = 0.70). A study by Suchting et al^34^ performed automatic VRA based on roughly 300 structured variables with comparable performance to our approach (AUC = 0.78).

The terms obtained in exploratory analysis, before application of modeling techniques, demonstrate a potential new type of risk factor that should be taken into account. Violence risk assessment instruments are often based on a combination of static factors (eg, previous violent behavior or employment status) and dynamic factors (eg, hostility or disorder symptoms). The terms we extracted from text are mostly dynamic and pertain to behavioral cues (eg, *angry* or *walked*) and social interactions (eg, *reacts* or *offered*), which may be more difficult to capture in a structured instrument but appear to provide important additional information.

A major strength of our research is the translational value that is obtained by using clinical notes from the EHR. Clinical text is already recorded as part of treatment by most psychiatric health care institutions, implying that our machine learning approach can be widely used to support violence management in daily practice. Second, applying a flexible machine learning approach allows method customization to local requirements and furthermore reveals the predictive validity for the relevant population, which is of particular importance given the lack of robustness and generalizability of existing models and methods. Finally, much attention has been devoted to the *actuarial* vs *clinical* debate,^35^ pertaining to the question of whether actuarial VRA instruments or VRA instruments based on clinical judgment are superior. Our approach essentially combines both approaches by using clinical judgment captured in clinical notes as input for an actuarial tool. This allows leveraging of health care professionals’ clinical experience while establishing a reasonably objective judgment through subsequent statistical modeling.

### Limitations

This study has limitations. One limitation is that the data obtained from EHRs were originally designated for treatment rather than research. This introduces some noise to our data set, in clinical notes and in violence incident reports, for example, in reporting discrepancies among different wards. This source of measurement uncertainty cannot be quantified, warranting some caution when interpreting our results. Furthermore, we predominantly used AUC, a measure of discrimination, to measure the predictive validity of our models. This measure is known to have some limitations, such as an inability to account for prevalence.^36^ We used a black box modeling approach combining the paragraph2vec and support vector machine algorithms to assess violence risk, inhibiting a straightforward substantiation of probability of violent behavior. Although the terms obtained in exploratory analysis together with the subgroup analysis of predictive validity have elucidated the problem context to some extent, they do not directly explain model behavior. How such explanations can reliably be obtained, both at the patient level and the model level, is still a topic of ongoing research in computer science.^37^ An exploration of model explainability is included in eAppendix 5 and the eFigure in the Supplement.

Before an automatic VRA approach can be used in practice, some important challenges need to be addressed. Our results point out that both high sensitivity and high specificity are unlikely to be achieved simultaneously. Further research is needed to point out the desired balance between false-positives and false-negatives, and hence, whether our prognostic models are most useful to identify patients at high or at low risk of violence. Additionally, what level of substantiation is necessary before automatic VRA can be used in practice also remains an open question, which should be addressed in discussion with professionals in the field.

## Conclusions

In the near future, we envision that further advancements toward a data-driven psychiatric practice will be made and that EHR data will become an even more valuable asset in supporting important decisions in the clinical process. Machine learning approaches have been able to contribute substantially in other fields of medicine, and our study provides evidence that such progress is possible in mental health care as well. Although some crucial challenges need to be addressed before adoption is possible, this study highlights the potential value of EHR data, and clinical notes in particular, for decision support. Such support systems may in the future be widely applied in daily practice, contributing to more effective and efficient psychiatric treatment.

## Notes

Supplement. **eAppendix 1.** Data Set Formation **eAppendix 2.** Paragraph2vec Model Training **eAppendix 3.** Cross-validation Procedure **eAppendix 4.** Code and Data Availability **eAppendix 5.** Model Explainability **eFigure.** Two Samples of Local Explanations of Models **eTable 1.** Chosen Paragraph2vec Model Settings **eTable 2.** Optimal Hyperparameters and Optimal AUC Based on the Inner Cross-validation Loop **eTable 3.** Subgroup Analysis of Model Performance: Early vs Late Violence **eTable 4.** Subgroup Analysis of Model Performance: Short vs Long Admissions **eReferences**
