# Supplementary material for: Machine Learning Approach to Inpatient Violence Risk Assessment Using Routinely Collected Clinical Notes in Electronic Health Records
Source: JAMA Netw Open. 2019 Jul 3;2(7):e196709. doi: 10.1001/jamanetworkopen.2019.6709 (PMC6613290; doi:10.1001/jamanetworkopen.2019.6709)
Supplement: Supplement. — eAppendix 1. Data Set Formation eAppendix 2. Paragraph2vec Model Training eAppendix 3. Cross-validation Procedure eAppendix 4. Code and Data Availability eAppendix 5. Model Explainability eFigure. Two Samples of Local Explanations of Models eTable 1. Chosen Paragraph2vec Model Settings eTable 2. Optimal Hyperparameters and Optimal AUC Based on the Inner Cross-validation Loop eTable 3. Subgroup Analysis of Model Performance: Early vs Late Violence eTable 4. Subgroup Analysis of Model Performance: Short vs Long Admissions eReferences [file jamanetwopen-2-e196709-s001.pdf]

Supplementary Online Content

Menger V, Spruit M, van Est R, Nap E, Scheepers F. Machine learning approach to inpatient violence risk assessment using routinely collected clinical notes in electronic health records. *JAMA Netw Open*. 2019;2(7):e196709. doi:10.1001/jamanetworkopen.2019.6709

eAppendix 1. Data Set Formation
eAppendix 2. Paragraph2vec Model Training
eAppendix 3. Cross-validation Procedure
eAppendix 4. Code and Data Availability
eAppendix 5. Model Explainability
eFigure. Two Samples of Local Explanations of Models
eTable 1. Chosen Paragraph2vec Model Settings
eTable 2. Optimal Hyperparameters and Optimal AUC Based on the Inner Cross-validation Loop
eTable 3. Subgroup Analysis of Model Performance: Early vs Late Violence
eTable 4. Subgroup Analysis of Model Performance: Short vs Long Admissions
eReferences

This supplementary material has been provided by the authors to give readers additional information about their work.

© 2019 Menger V et al. *JAMA Network Open.*

eAppendix 1: Data Set Formation

Our study makes extensive use of data that is routinely collected in patients' Electronic Health Records (EHRs). A major advantage of this data is that it has already been collected for the sake of delivering quality treatment, and can be made available for retrospective research designs in many cases. This advantage is simultaneously its major weakness: since this data lacks a study protocol to guarantee adequate measurements, important data quality challenges need to be addressed. High effort is associated with unlocking this type of data for research. Before any modeling can commence, several issues need to be resolved, for example regarding data extraction (i.e. obtaining relevant data entities from EHR systems), data provenance (i.e. tracking data points to their origin in the EHR), and data preparation (i.e. applying appropriate transformations to data). If the goal of a study using retrospective EHR data is to obtain new insights with confidence, serious attention needs to be devoted to these steps.

In the Department of Psychiatry of the University Medical Center Utrecht (site 1), we initiated the Psydata Project in 2015, with the goal to improve care in daily practice by obtaining new insights and decision support through analysis of retrospective EHR data. After proving feasibility of such a project[1], we set out to structurally address challenges such as mentioned above (data extraction, data provenance, data preparation) in order to learn from EHR data. Since this is a relatively new area of research, we solved this problem by developing the Capable Reuse of EHR data (CARED) framework[2]. We identified the most important challenges of reusing EHR data and then proposed the framework for infrastructure that can address them. A technical infrastructure based on this framework was implemented, to support our EHR data analysis goals. This technical infrastructure for example addresses reproducibility of research, data preparation, collaboration among researchers, and documentation of code and data. Together with organizational artefacts such as guidelines for documentation and internal control, this can guarantee data quality. Our current practices ensure an up-to-date, de-identified, and accessible dataset of most information that is recorded in the EHR. The system is maintained by a multidisciplinary team of professionals, that documented the process and data in detail. The dataset used for this study, consisting of information about admissions, incidents and clinical notes, is thus a result of careful deliberation among both data analysts and practitioners, securing its validity.

At Antes (site 2), EHR data are extracted to a clinical data warehouse that is designed largely in line with the requirements of CARED. Given the goal of attempting to replicate findings in site 1, for defining the cohort and selecting the data we followed choices that were mandated by the study design in site 1. Where knowledge specific to this site was involved (e.g. in selecting the appropriate wards), extra attention was devoted to consult with local experts. Choices in both sites were finally discussed in a focus group with stakeholders from both sites present, in order to check whether any discrepancies between choices in both sites existed. No such discrepancies were identified during the meeting, guaranteeing a similar dataset with the same standard for data quality.

© 2019 Menger V et al. *JAMA Network Open.*

eAppendix 2: Paragraph2vec Model Training

Since classification models use numbers as input rather than text, a suitable vector representation of clinical notes is needed before classification can occur. For this purpose we have used the paragraph2vec algorithm[3], which is an extension of the earlier word2vec algorithm[4]. Both algorithms operate on the principle of learning a vector representation of arbitrary dimensionality using a large corpus of relevant text. This is achieved by training a neural model with a hidden layer to predict target words (i.e. a word in a sentence) based on its context words (i.e. its surrounding words). The learning process takes place in an unsupervised way, meaning that no outcome variable or document labels are needed to learn accurate vector representations. The word2vec algorithm produces a corresponding vector in the vector space for each word in the training corpus. It's main advantage over a simple bag-of-words approach is that word2vec vector representations allow vector operations such as addition, subtraction and cosine similarity, that can produce semantically meaningful results. The paragraph2vec algorithm produces a corresponding vector for each document in the training corpus, and additionally also allows inferring vectors for unseen texts. This is a probabilistic process, that works by fixing the weights of the neural model and optimizing a randomly initialized representation vector, rather than the other way round during representation training.

Since clinical text is a domain-specific language that can contain idiosyncrasies, spelling errors, and terms that have domain-specific meanings, pre-trained paragraph2vec models that are for instance trained on Wikipedia or Google News data do not necessarily yield useful representations for clinical notes. For this reason, in both sites we obtained a large internal set of de-identified clinical notes, both with at least 1 million notes, to train paragraph2vec models. As preprocessing steps, we transformed all text to lowercase, remapped special characters, and removed all characters that were not whitespace, period or alphabetical characters. We then tokenized text (i.e. split it to words), removed stop words, and applied stemming (i.e. mapping inflections of words to their stem). The resulting sequence of terms was then used to train a paragraph2vec model. Optimal paragraph2vec model settings are still a topic of ongoing research, we based our choices on default model settings in the Gensim[5] package that was used for training, in combination with information by Chiu et al. and Lau et al.[6,7] (eTable 1). We used the Distributed Memory model for training the algorithm, which concatenates input vectors and is thus able to take word order into account. Model dimensionality typically ranges between 100 and 1000, we opted for a dimensionality of 300 as a middle ground. We slightly decreased the window size from 5 to 2, and increased the minimum word count from 5 to 20 to mitigate effects of lack of structure and spelling errors present in clinical text. We increased the number of epochs to 20, in order to increase the likeliness of reaching model convergence on our data set. Other parameters were not changed from Gensim defaults. The result of training includes two independent paragraph2vec models that comprise the machine learning pipeline together with the classification models.

In order to determine numerical representations of clinical notes in our dataset using the trained paragraph2vec model, we first concatenated all relevant notes for a single admission, and then averaged over ten paragraph2vec inferences of this unseen concatenation of notes, to cancel out inaccuracies due to the probabilistic nature of the inference.

eAppendix 3: Cross-validation Procedure

When applying machine learning models to a dataset, one must ensure that data is never simultaneously used to train and test a model. Information leakage between these two sets will inevitably lead to overly optimistic estimates of model predictive validity. We chose a nested cross validation procedure, to simultaneously optimize, train and assess the predictive validity of a model on a single dataset while obtaining a reliable estimate of performance without bias.

Our classification model consists of a Support Vector Machine with a radial kernel. This type of machine learning algorithm has two hyperparameters that should be optimized: the cost parameter (C) that determines how strong models during training are penalized for data points on the wrong side of the classification boundary, and the gamma (ϒ) parameter that determines how far the influence of a single training example reaches. We determined the optimal values for these parameters using a grid search, i.e. by training a Support Vector Machine for multiple combinations of C and ϒ values. For C we chose a range of $[10^{-1}, 10^{0}, 10^{1}]$, and for ϒ we chose $[10^{-6}, 10^{-5}, \ldots, 10^{0}]$. We chose a relatively narrow range for C because models trained on our dataset are empirically not very sensitive to this parameter, and to speed up model training time. Model performance was then estimated on a hold-out set, i.e. a subset of data that is not used for training models. Since using one single hold-out set can introduce bias into performance estimates, we used cross validation to repeat this process five times on non-overlapping test sets, and chose hyperparameters that perform best on average. This procedure comprises the inner cross validation loop CV_{inner}.

A new model was then trained using data of all five CV_{inner} folds, using the optimal hyperparameters found in the CV_{inner} loop, and performance was tested on yet another hold-out set. For the same reasons as mentioned above, we repeated this procedure in five folds as well, in the CV_{outer} cross validation. While the CV_{inner} loop is used to determine optimal hyperparameters, the CV_{outer} loop is used obtain a reliable estimate of performance on unseen data. In both cross validation loops, we furthermore ensured that datapoints from the same patients (i.e. previous or future admissions) were always grouped in the same fold, mainly to prevent information of future admissions influencing performance assessment.

Given the five folds $\text{test}^1_{\text{outer}}, \ldots, \text{test}^5_{\text{outer}}$ that were used to estimate performance in CV_{outer}, we computed the Area Under Curve by averaging over the five folds, i.e. using $\text{AUC} = \frac{1}{5}\sum_{i=1}^{5} \text{AUC}(\text{test}^i_{\text{outer}})$. To estimate standard error of the mean of AUC, we used the DeLong method[8] for estimating variance of AUC for each fold using $\text{VAR}^i = \text{delong-var}(\text{test}^i_{\text{outer}})$. The DeLong method is applicable in this case, and preferred when other methods based on bootstrapping are computationally not feasible[9]. We then computed average variance (VAR) over the five folds $\text{VAR} = \frac{1}{5}\sum_{i=1}^{5} \text{VAR}^i$, and took the square root to compute the average standard deviation $\text{SD} = \sqrt{\text{VAR}}$. To estimate the standard error of the mean AUC we finally used $\text{SE} = \text{SD}/\sqrt{5}$, given AUC samples in five different folds. Other outcome statistics were determined based on a 2x2 contingency table, showing true negatives, false negatives, false positives and true positives. To map classification probabilities (i.e. probability of showing violent behavior) to a binary outcome, we set a classification threshold so that classification has the same distribution as outcome (i.e. the true labels). This ensures false positives and false negatives are balanced, as the optimal balance for daily practice still needs to be established. The classification threshold was set per fold, because predictions among different folds are not necessarily calibrated with regard to each other. The contingency table was finally determined by summing per-fold contingency tables, and other statistics such as sensitivity and specificity are determined based on this contingency table.

Results of the hyperparameter optimization procedure are displayed in eTable 2. The Area Under Curve (AUC) values are based on internal cross validation loop. These values are relatively close to outer cross validation results, showing that model convergence has been reached, while the cross validation setup has inhibited overtraining of models.

© 2019 Menger V et al. *JAMA Network Open.*

eAppendix 4: Code and Data Availability

We have made all analysis code, predictions, and output logs available in an online GitHub repository. This allows other researchers to verify that analysis was indeed performed as described in this paper, makes data accessible for meta studies and allows potential reproduction of our results by other researchers on new and independent datasets.

The analysis code is available in the form of a set of Jupyter Notebooks, which implement Python scripts developed for this study. Predictions and output logs are available in csv and text files. The GitHub repository is accessible at the following link, where additional information and documentation can be found: https://www.github.com/vmenger/violence-risk-assessment.

All data associated with the study is stored internally, so that results can be verified and modifications of method or potentially new modeling techniques can be applied in the future. Unfortunately, the datasets cannot be shared with other researchers due to legal and privacy constraints.

eAppendix 5: Model Explainability

In order to explore whether classification model behavior at the local level can be explained, we applied the Linear Model-Agnostic Explanations (LIME) method[10] to our trained models. This method tries to approximate the decision boundary near a specific data point using a linear function. Specifically, it samples points around a data point that is to be explained, and uses the trained machine learning pipeline to classify this set of data points. Based on these data points and their classified outcome, LIME trains a k-lasso model on a bag-of-words representation of sampled data points, returning a set of k terms in these texts that are relevant for the local decision boundary.

Based on an exploratory evaluation that presented explanations of a small subset of data points to eight human subjects, we found that presenting an explanation (e.g. eFigure 1) in combination with a risk assessment increased participants' trust in the system. We additionally found no evidence of bias (e.g. discrimination against protected groups) in the classification model. Some points of model failure were finally identified, where texts were classified using, apparently to the human user, arbitrary terms. This information can be used as feedback to improve the dataset and trained models.

© 2019 Menger V et al. *JAMA Network Open.*

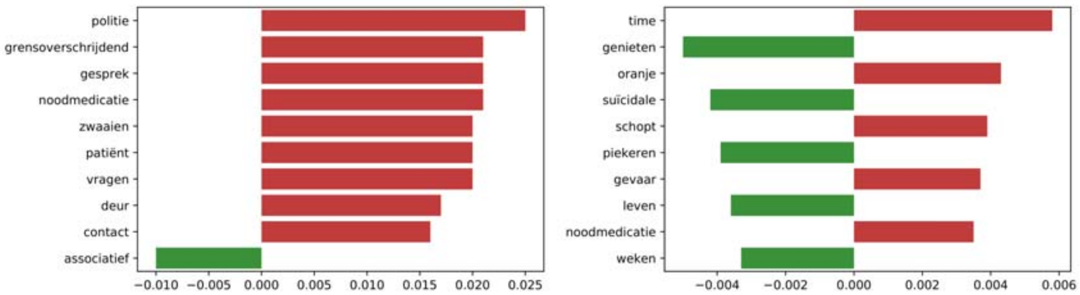

eFigure. Two Samples of Local Explanations of Models

The explanation on the left predicted high risk of aggression, which is reflected in terms such as *politie* (‘police’) and *noodmedicatie* (‘emergency medication’). The explanation on the right predicted low risk, explained by terms such as *genieten* (‘enjoy’) and *suïcidale* (‘suicidal’), but also exhibits high-risk terms such as *schopt* (‘kicks’) and *gevaar* (‘danger’).

© 2019 Menger V et al. *JAMA Network Open.*

eTable 1. Chosen Paragraph2vec Model Settings

**Parameter**	**Value**
Batch size	10,000
Epochs	20
Learning rate	0.025 – 0.001
Learning rate decay	Linear
Minimum count	20
Model	Distributed Memory
Sub sampling	0.001
Vector size	300
Window size	2

© 2019 Menger V et al. *JAMA Network Open.*

eTable 2. Optimal Hyperparameters and Optimal AUC Based on the Inner Cross-validation Loop

Fold	Site 1				Site 2			
	**C**	**ϒ**	**Inner AUC (SD)**	**Outer AUC**	**C**	**ϒ**	**Inner AUC (SD)**	**Outer AUC**
1	0.1	0.001	0.797 (0.008)	0.755	1.0	0.01	0.753 (0.044)	0.761
2	0.1	0.001	0.792 (0.038)	0.813	1.0	0.01	0.759 (0.029)	0.741
3	0.1	0.001	0.784 (0.019)	0.805	10.0	0.01	0.742 (0.044)	0.784
4	1.0	0.0001	0.790 (0.038)	0.792	1.0	0.01	0.741 (0.039)	0.782
5	0.1	0.001	0.786 (0.040)	0.818	1.0	0.01	0.762 (0.038)	0.752

A grid search is performed to determine the optimal support vector machine parameters. The Inner AUC column shows the average AUC over five inner folds, while the Outer AUC column specifies performance on the hold-out fold for this iteration. Abbreviations: AUC = Area Under Curve, SD = Standard Deviation, C = cost, ϒ = inverse of kernel radius.

© 2019 Menger V et al. *JAMA Network Open.*

eTable 3. Subgroup Analysis of Model Performance: Early vs Late Violence

Evaluation	Day of first violence incident (median)	Early violence AUC (95% CI)	Late violence AUC (95% CI)	Difference (95% CI)	P-value
Internal (CV), site 1	6	0.821 (0.787 to 0.854)	0.775 (0.740 to 0.810)	0.046 (-0.003 to 0.094)	0.06
Internal (CV), site 2	9	0.771 (0.724 to 0.818)	0.755 (0.708 to 0.803)	0.015 (-0.051 to 0.082)	0.65
External model, site 1	6	0.745 (0.704 to 0.785)	0.698 (0.652 to 0.744)	0.046 (-0.015 to 0.108)	0.14
External model, site 2	9	0.653 (0.609 to 0.698)	0.632 (0.587 to 0.678)	0.021 (-0.042 to 0.085)	0.51

Abbreviations: CI = Confidence Interval, CV = Cross Validation.

© 2019 Menger V et al. *JAMA Network Open.*

eTable 4. Subgroup Analysis of Model Performance: Short vs Long Admissions

Evaluation	Length of admission (median)	Short admissions AUC (95% CI)	Long admissions AUC (95% CI)	Difference (95% CI)	P-value
Internal (CV), site 1	16	0.805 (0.764 to 0.846)	0.792 (0.758 to 0.826)	0.012 (-0.041 to 0.066)	0.65
Internal (CV), site 2	15	0.789 (0.738 to 0.839)	0.730 (0.686 to 0.774)	0.058 (-0.008 to 0.125)	0.09
External model, site 1	16	0.704 (0.653 to 0.755)	0.736 (0.700 to 0.775)	0.032 (-0.033 to 0.096)	0.34
External model, site 2	15	0.655 (0.607 to 0.702)	0.633 (0.589 to 0.678)	0.022 (-0.043 to 0.087)	0.52

Abbreviations: CI = Confidence Interval, CV = Cross Validation

© 2019 Menger V et al. *JAMA Network Open.*

eReferences

1. Menger V, Spruit M, Hagoort K, Scheepers F. Transitioning to a Data Driven Mental Health Practice: Collaborative Expert Sessions for Knowledge and Hypothesis Finding. *Comput Math Methods Med*. 2016;2016. doi:10.1155/2016/9089321.
2. Menger V, Spruit M, de Bruin J, Kelder T, Scheepers F. Supporting Reuse of EHR Data in Healthcare Organizations: the CARED Research Infrastructure Framework. In: *Proceedings of the 12th Conference on Health Informatics*. ; 2019:41-50.
3. Le Q, Mikolov T. Distributed Representations of Sentences and Documents. In: *Proceedings of the 31st International Conference on International Conference on Machine Learning - Volume 32*. ; 2014:II-1188-II-1196.
4. Mikolov T, Sutskever I, Chen K, Corrado G, Dean J. Distributed Representations of Words and Phrases and Their Compositionality. In: *Proceedings of the 26th International Conference on Neural Information Processing Systems - Volume 2*. ; 2013:3111-3119.
5. Řehůřek R, Sojka P. Gensim - Statistical Semantics in Python. In: *EuroScipy*. ; 2011. doi:10.3200/SOCP.146.2.250-252.
6. Chiu B, Crichton G, Korhonen A, Pyysalo S. How to Train good Word Embeddings for Biomedical NLP. In: *Proceedings of the 15th Workshop on Biomedical Natural Language Processing*. ; 2016:166-174. doi:10.18653/v1/W16-2922.
7. Lau JH, Baldwin T. An Empirical Evaluation of doc2vec with Practical Insights into Document Embedding Generation. https://arxiv.org/pdf/1607.05368.pdf. Accessed April 12, 2018.
8. DeLong ER, DeLong DM, Clarke-Pearson DL. Comparing the Areas under Two or More Correlated Receiver Operating Characteristic Curves: A Nonparametric Approach. *Biometrics*. 1988;44(3):837-845. doi:10.2307/2531595.
9. Hajian-Tilaki KO, Hanley JA. Comparison of three methods for estimating the standard error of the area under the curve in ROC analysis of quantitative data. *Acad Radiol*. 2002;9:1278-1285. doi:10.1016/S1076-6332(03)80561-5.
10. Ribeiro MT, Singh S, Guestrin C. "Why Should I Trust You?" In: *Proceedings of the 22nd ACM SIGKDD International Conference on Knowledge Discovery and Data Mining - KDD '16*. ; 2016. doi:10.1145/2939672.2939778.

© 2019 Menger V et al. *JAMA Network Open.*
